# Upregulation of necroptosis markers RIPK3/MLKL and their crosstalk with autophagy-related protein Beclin-1 in primary immune thrombocytopenia

**DOI:** 10.1007/s10238-022-00839-8

**Published:** 2022-06-14

**Authors:** Amany M. Kamal, Nermeen A. Nabih, Nahed M. Rakha, Eman F. Sanad

**Affiliations:** 1 Department of Biochemistry and Molecular Biology, Faculty of Pharmacy, Ain Shams University, African Union Organization Street, Abassia, 11566 Cairo Egypt; 2 Internal Medicine Department, Clinical Hematology and Bone Marrow Transplantation Unit, Faculty of Medicine, Ain Shams University, Cairo, Egypt

**Keywords:** Platelets, Primary immune thrombocytopenia, Necroptosis, MLKL, RIPK3, Beclin-1

## Abstract

Necroptosis is a novel form of programmed necrotic cell death involved in various autoimmune diseases. The potential role of necroptosis in primary immune thrombocytopenia (ITP) and the possible interlink with autophagy have not been fully investigated. The gene expression of mixed lineage kinase-like domain (MLKL), receptor-interacting protein kinase 3 (RIPK3) and Beclin-1 were quantified in peripheral blood of 45 ITP patients and 20 healthy controls. Their associations with clinical, laboratory parameters and response to steroid therapy in ITP patients were evaluated. RIPK3, MLKL, and Beclin-1 were significantly upregulated in ITP patients than in healthy controls (*P* < 0.001). Beclin-1 mRNA levels were positively correlated with both RIPK3 and MLKL mRNA levels in ITP patients (*P* < 0.0001). In addition, MLKL, RIPK3, and Beclin-1 mRNA levels were inversely correlated with platelet count (*r* = −0.330, −0.527 and −0.608, respectively). On the hand, positive correlations between MLKL (*P* = 0.01), RIPK3 (*P* = 0.005), Beclin-1 (*P* = 0.002) mRNA levels and severity of bleeding in ITP patients were reported. Steroid responders (*n* = 18, 40%) had significantly lower MLKL, RIPK3, Beclin-1 mRNA expression levels than their levels in the non-responders (*n* = 27, 60%). Necroptosis may play a critical role in the pathogenesis of ITP and provide both novel therapeutic targets and promising biomarkers for the prediction of bleeding severity and treatment response in ITP patients. Additionally, this study highlighted the crosstalk between autophagy and necroptosis in ITP patients.

**Graphical abstract:**

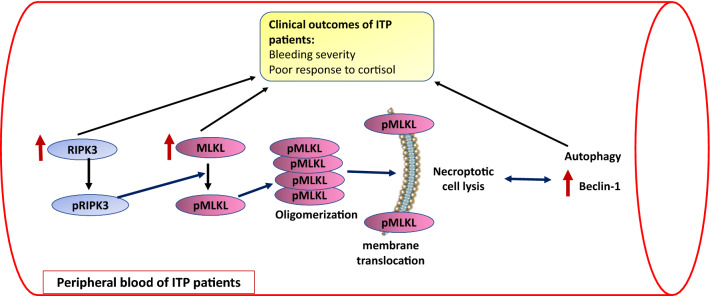

## Introduction

Cell death is a fundamental process that regulates the cells’ life span and maintains tissue homeostasis [1]. Nevertheless, inappropriate cell death either excessive or insufficient is linked to the etiology of numerous illnesses including cancer, atherosclerosis and autoimmune disorders [2]. Previously, it was believed that there were only three forms of cell death, apoptosis, autophagy and necrosis. Apoptosis and autophagy are two different forms of programmed cell death, while necrosis is uncontrolled lytic cell death in reaction to external stressful stimuli [3].

Research has dramatically changed the previous paradigm of cell death with the discovery of necroptosis and pyroptosis. They are lytic inflammatory pathways of programmed cell death that have the capacity to trigger inflammatory responses via the release of damage-associated molecular patterns (DAMPs) and secretion of inflammatory cytokines such as interleukin (IL)-1β and IL-18 [4–6]. Pyroptosis was first described in 1992 but the term was coined in 2001 [7]. Although it was traditionally known as caspase-1-mediated cell death, several apoptotic effector caspases like caspase 11, 4/5 and 3 can trigger pyroptosis [5, 6, 8]. On the other hand, necroptosis is a type of programmed necrosis which occurs when apoptosis is inhibited [9, 10]. Necroptosis is morphologically like necrosis but distinctive from apoptosis however both necroptosis and apoptosis shared the same range of triggering factors [3, 11].

Tumor necrosis factor (TNF) is considered the most important trigger for necroptosis. In brief, the molecular mechanism of necroptosis started with stimulation of TNF receptor with subsequent activation of receptor-interacting protein kinase (RIPK) 1 and 3. This leads to the formation of a microfilament complex called necrosome which activates mixed lineage kinase-like domain (MLKL), the executor of necroptosis. Activated MLKL oligomerizes and translocates to plasma membrane inducing membrane damage and cell lysis which causes the release of cellular contents as DAMPs eliciting an immune response [11, 12]

The dominant role of necroptosis in the development of several diseases associated with unjustified cell loss and inflammatory responses such as multiple sclerosis, psoriasis, rheumatoid arthritis, myocardial infarction, inflammatory bowel disease and some malignant diseases has been reported [13, 14]. Therefore, necroptosis-based therapy is the focus of much ongoing advanced research work.

Primary immune thrombocytopenia (ITP), a common acquired immune-mediated bleeding disorder, is characterized by unexplained isolated thrombocytopenia (low platelet count of < 100 × 10^9^/L) hence diagnosed by exclusion [15, 16]. Frontline treatment of ITP includes immune suppression with a short course of corticosteroids while splenectomy, rituximab and thrombopoietin receptor agonists are considered the second-line treatment [17]. Despite the availability of different lines of treatment for this benign hematological disorder, patients have poor life quality due to recurrent bleeding events, frequent exacerbations of disease activity (relapse) and treatment-related complications [18]

Pathogenesis of ITP is heterogeneous, multifactorial and quite complex but to date much about it remains not fully elucidated [19, 20]. While traditionally the hallmark of ITP pathogenesis was enhanced antibody-mediated platelet phagocytosis, recent research proved compelling evidence that several other immune-mediated abnormalities have been involved such as autoantibody-mediated destruction of megakaryocytes in the bone marrow. In addition, ITP is characterized by numerous T-cell mediated immune dysregulations such as downregulation of regulatory T-cells (Tregs), induction of abnormal T-helper (Th)1 cells associated with loss of Th1/Th2 balance and activated cytotoxic T-cells that mediate platelets destruction and apoptosis of megakaryocytes [21, 22]. ITP patients demonstrate a Th-type cytokine bias that is characterized by elevated serum levels of Th17-associated cytokines (IL-17 and IL-6) and Th1-associated cytokines (IL-2 and interferon gamma) [23, 24].

Dysregulated programmed cell death pathways are closely linked to the pathogenesis of ITP [25]. The contribution of autophagy and abnormal apoptosis of platelet and/or megakaryocytes were found to be participated in the ITP pathogenesis and correlated to decreased production and life span of platelet [26–28]. Also, Olsson *et al*. described defective apoptosis in T cells from patients with ITP [29]. Previous studies have confirmed close relation between apoptosis and autophagy in physiological and pathological states while studies that investigated the interconnection between necroptosis and autophagy revealed conflicting results [30–32]. The exact role of necroptosis in ITP and its crosstalk with autophagy has not been investigated before.

Herein, we explored the expression pattern of necroptosis-related markers RIPK3/MLKL along with autophagy-related protein Beclin-1 in ITP patients and assessed their associations with clinicopathological characteristics of ITP patients. We also evaluated their diagnostic utility in discriminating between responders and non-responders to cortisol medication.

## Subjects and methods

### Participants

The current study comprised 45 patients newly diagnosed with ITP (40 females and 5 males; mean age of 35.6 ± 1.8 years). They were recruited from the Internal Medicine Department at Ain-shams University Hospitals, Cairo, Egypt, from January 2020 to February 2021. All patients met the current international diagnostic criteria of ITP [33]. The diagnosis was based on the patient medical history, physical examination with emphasis on evaluation of bleeding score according to ITP bleeding scale (IBSL), (Grade 0; no bleeding, Grade 1; mild bleeding, Grade 2; severe bleeding manifestation) [34]. The laboratory investigations included complete blood count, peripheral blood smear, bone marrow smears and other investigations were used to exclude secondary ITP (immunological markers, virology screen for HCV, HBV and HIV). All included patients had primary ITP and did not receive any medical treatment before sampling. Patients with primary ITP who received treatment or patients with secondary ITP were excluded from the current study. All enrolled patients required treatment either because of clinically significant bleeding or due to platelet count less than 30 × 10^9^/L, the initiation and choice of first-line treatment were according to ASH 2019 guidelines for immune thrombocytopenia [35]. They received a short course of oral prednisone 1 mg/kg/day in tapering doses for 4–6 weeks. Follow-up of response to treatment after 4 weeks with complete blood count, complete response was defined as platelet count > 100 × 10^9^/L measured on 2 occasions with 7 days apart according to International Work Group of ITP [36]. The control group included 20 healthy age and sex-matched participants (17 females and 3 males; mean age of 33 ± 1.3 years).

Each enrolled participant signed an informed consent before study entry. This study adheres to the principles of the Declaration of Helsinki and was approved by the Ethical Committee of Research, Faculty of Medicine, Ain Shams University.

### Blood sampling

Peripheral blood samples were obtained from each adult ITP patient (before initiation to treatment) and healthy controls. Blood samples were collected on vacutainer tubes containing ethylenediamine tetra-acetic acid, disodium salt (Na_2_ EDTA) and immediately transferred to the laboratory for blood processing. Whole blood was used to determine the complete blood count and for total RNA isolation.

### RNA isolation and quantitative real time-PCR assays

About 1.5 mL of whole blood was used to extract total RNA using spin column technology of QIAamp RNA Mini Kit supplied by Qiagen (USA). The concentration and purity of extracted RNA were evaluated by Platinum-colored DS11 Spectrophotometer (DeNovix Inc, USA). The High-Capacity cDNA Reverse Transcription Kit (catalog number; 4374967) supplied by Thermo Fisher Scientific (USA) was used for complementary DNA (cDNA) synthesis. For each reaction tube, 10 µL of prepared 2 × Reverse Transcriptase (RT) master mix and 500 ng RNA template were added and the nuclease-free water adjusted the final volume at 20 µL. The cDNA synthesis was performed by Techne TC-3000G Thermal Cycler (San Diego, CA, USA) and the transcription protocol was adjusted for 10 min at 25℃ followed by 120 min at 37℃ then 5 min at 85 °C. The cDNA was stored at − 20 °C until quantification of target genes.

Quantitative PCR (QPCR) was carried out using predesigned ready-to-use primers and probe TaqMan™ gene expression assays for target genes (human MLKL, RIPK3 and Beclin-1) and housekeeping gene; glyceraldehyde 3-phosphate dehydrogenase (GAPDH) provided by Thermo Fisher Scientific (USA). Table 1 illustrates the specific TaqMan assay ID, assay design, dye used and amplicon size of each gene. Each TaqMan™ gene expression assay contains 18 µM of corresponding forward and reverse primers and 5 µM of the specific probe in 20 × format. TaqMan gene expression was performed on Step One PCR detection system (Applied Biosystems, USA) in a reaction containing 50 ng cDNA, 10 µL TaqMan™ Gene Expression Master Mix (catalog no. 4370048), 1 µL of corresponding TaqMan™ gene assays in a final volume of 20 µL. The reaction profile used was as follows 2 min at 50 °C for uracil-N-glycosylase activation, 10 min at 95 °C followed by 40 PCR cycles of denaturing (15 s at 95 °C) and annealing/extension (1 min at 60 °C). Negative control was included in each run. The expression of target genes was normalized to the expression of the internal reference (GAPDH). Relative gene expression was calculated and normalized as fold-change using the CT cycle method (2^−∆∆Ct^) where ΔΔCt = (Ct_target_ gene–Ct_GAPDH_) _patient_—(Ct_target gene_—Ct_GAPDH_) _control_ [37].

**Table 1:** The specific TaqMan assay ID, assay design, dye used and amplicon size of each gene

Gene	Entrez Gene ID	TaqMan Assay No	Assay design	Amplicon size	Dye
MLKL	197259	Hs04188505_m1	probe spans exon	90	FAM-MGB
RIPK3	11035	Hs00179132_m1	probe spans exon	70	FAM-MGB
Beclin1	8678	Hs01007018_m1	probe spans exon	54	FAM-MGB
GAPDH	2597	Hs02786624_g1	both assay primers and probes lie within a single exon	157	VIC-MGB

*FAM* fluorescein amidites; *GAPDH* glyceraldehyde 3-phosphate dehydrogenase; *MGB* minor groove binder; *MLKL* mixed-lineage kinase domain-like; *RIPK3* receptor-interacting protein kinase 3

### Statistical analysis

Statistical analysis was executed by the Statistical Package for Social Science (SPSS) version 23 (IBM^©^ Corp., NY). The quantitative data were presented as mean ± standard error of means (SEM) while qualitative variables were presented as numbers and percentages. Differences between groups were compared using the Mann–Whitney U-test and student’s T test for nonparametric and parametric continuous variables, respectively*.* The qualitative data were analyzed by using the Chi-square *test.* Correlations between continuous variables were tested using the Spearman rank correlation, while point biserial correlation (r_bp_) was used when one of the variables was dichotomous. Receiver operating characteristic curve (ROC) was used to evaluate the utility of using necroptosis markers and Beclin-1 as a tool to discriminate between responders and non-responders to cortisol treatment. Two-sided *P* < 0.05 was considered significant.

## Results

### Characteristics of study populations

The demographic and clinical characteristics of the studied groups are summarized in Table 2.

**Table 2:** Demographic data, clinicopathological parameters and biochemical parameters levels of the studied groups

	ITP (*n* = 45)	Control group (*n* = 20)
Age (years)	35.62 ± 1.82	33.00 ± 1.38
Gender (Females/males)	40/5	17/3
Family history; n (%)	1 (2.2)	0 (0)
Bleeding scale (IBSL)		
Mild bleeding	15 (33.3%)	–
Severe bleeding	30 (66.7%)	–
Total leukocytes (10^3^ cells/cm^3^)^¶^	7.36 ± 0.38	6.48 ± 0.23
Hemoglobin (gm/dL)^¶^	9.35 ± 0.33^a^	13.28 ± 0.14
Platelet count (10^3^/cm^3^)^¶^	19.96 ± 3.78^a^	274.05 ± 9.18
Degree of thrombocytopenia		
Moderate	13 (28.9%)	–
Severe	32 (71.1%)	–
mRNA MLKL levels (fold expression)^¶^	3.31 ± 0.48^a^	1.00 ± 0.04
mRNA RIPK3 levels (fold expression)^¶^	2.87 ± 0.35^a^	1.01 ± 0.04
mRNA Beclin-1 levels (fold expression)^¶^	3.94 ± 0.46^a^	1.00 ± 0.03

Data are presented as mean ± SEM or numbers of patients (percentages), a considered statistically significant from control group at *P* ≤ 0.001

*MLKL* mixed-lineage kinase domain-like; *RIPK3* receptor-interacting protein kinase 3

^**¶**^Variables were analyzed by Mann–Whitney test

### Expression levels of RIPK3 and MLKL and autophagy-related gene (Beclin-1) in ITP patients

**Fig. 1:**
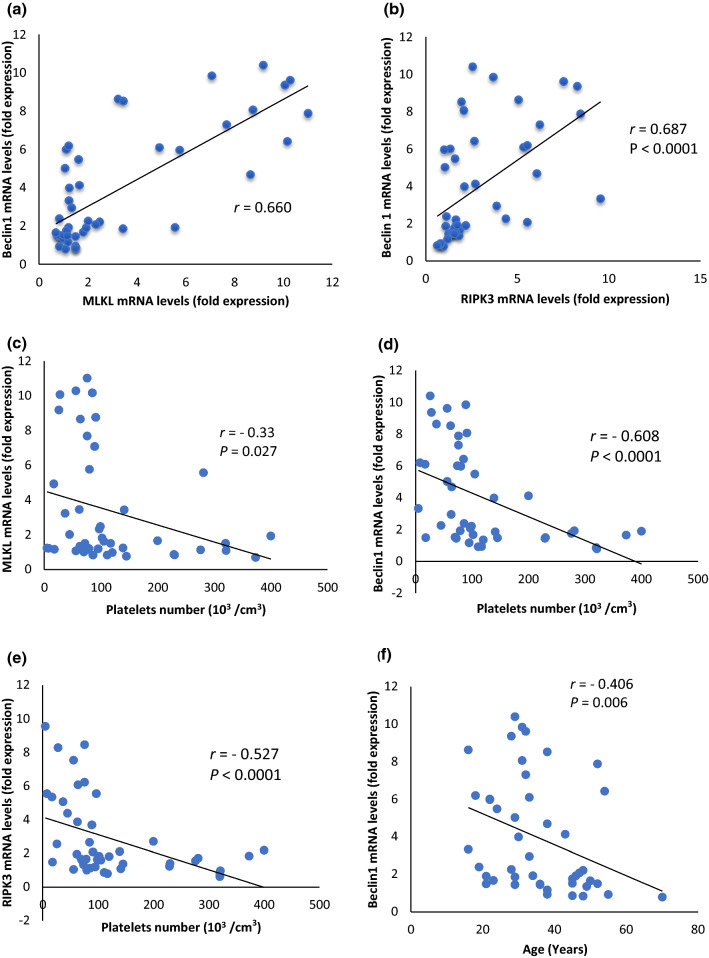
Correlation analysis of the studied parameters in ITP patients (*n* = 45). **a** Correlation between Beclin-1 mRNA level and MLKL mRNA level. **b** Correlation between Beclin-1 mRNA level and RIPK3 mRNA level. **c** Correlation between MLKL mRNA level and platelet count. **d** Correlation between Beclin-1 mRNA level and platelet count. **e** Correlation between RIPK3 mRNA level and platelet count. **f** Correlation between Beclin-1 mRNA level and age**.** MLKL, mixed-lineage kinase domain-like; RIPK3, receptor-interacting protein kinase 3

The mean expression levels of RIPK3, MLKL and Beclin-1 mRNA were significantly upregulated in ITP patients (2.87, 3.31 and 3.94, respectively) compared with their levels in healthy control subjects (1.00, 1.01 and 1.00, respectively) at *P* < 0.001 (Table 2). Moreover, there was a positive correlation between Beclin-1 mRNA levels and both RIPK3 (*r* = 0.687) and MLKL (*r* = 0.660) mRNA levels in ITP patients at *P* < 0.0001 (Fig. 1a, b).

### Analysis of MLKL, RIPK3, Beclin-1 expression levels and clinicopathological features of ITP patients

**Fig. 2:**
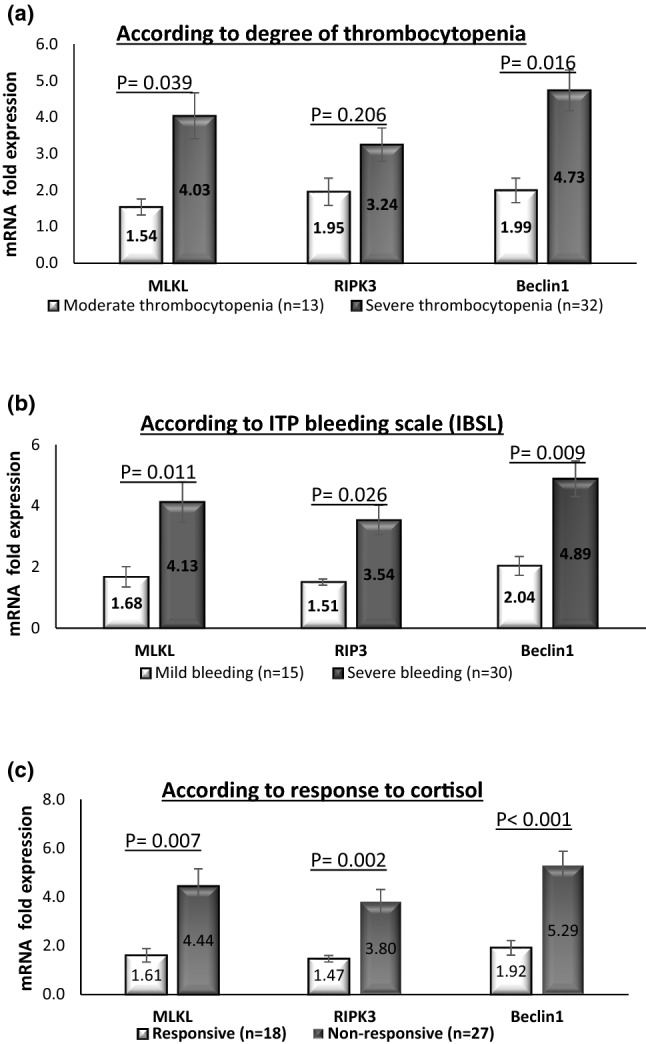
MLKL, RIPK3 and Beclin-1 mRNA expression levels in ITP patients according to **a** the degree of thrombocytopenia, **b** ITP bleeding scale (IBSL), **c** response to cortisol treatment. Data are expressed as mean ± SEM and analyzed by Mann–Whitney test. MLKL, mixed-lineage kinase domain-like; RIPK3, receptor-interacting protein kinase 3

MLKL, RIPK3 and Beclin-1 mRNA expression levels showed negative correlations with platelet count in ITP patients (*r* = −0.330, −0.527 and −0.608, respectively) as illustrated in Fig. 1c, d and e. Results demonstrated positive correlations between the degree of thrombocytopenia and both mRNA levels of MLKL (*r*_pb_ = 0.351; *P* = 0.018) and Beclin-1 (*r*_pb_ = 0.418; *P* = 0.004) while the correlation between RIPK3 expression level and degree of thrombocytopenia was not significant (*r*_pb_ = 0.250; *P* = 0.097) (Table 3). Patients with severe thrombocytopenia displayed significantly higher expression levels of MLKL and Beclin-1 than those who had moderate thrombocytopenia (4.03 ± 0.63 and 4.73 ± 0.56 vs 1.54 ± 0.22 and 1.99 ± 0.33, respectively) (Fig. 2a). Differences in MLKL (*P* = 0.01), RIPK3 (*P* = 0.02), Beclin-1 (*P* = 0.009) mRNA levels between patients with mild bleeding compared to patients with severe bleeding were significant (Fig. 2b). Moreover, there were positive correlations between MLKL (*r*_pb_ = 0.360; *P* = 0.01), RIPK3 (*r*_pb_ = 0.413; *P* = 0.005), Beclin-1 (*r*_pb_ = 0.453; *P* = 0.002) mRNA levels and severity of bleeding in ITP patients (Table 3).

**Table 3:** Correlation between MLKL, RIPK3 or Beclin-1 mRNA levels and clinicopathological parameters in ITP patients (*n* = 45)

	Correlation	mRNA MLKL levels	mRNA RIPK3 levels	mRNA Beclin1 levels
Age	Spearman’s rho *r*	−0.120	−0.199	−0.406**
	*P*	0.433	0.191	0.006
Total leucocyte count (10^3^cells/cm^3^)	Spearman’s rho *r*	−0.047	−0.080	−0.070
	*P*	0.758	0.777	0.647
Hemoglobin (gm/dL)	Spearman’s rho *r*	−0.024	−0.080	−0.114
	*P*	0.874	0.602	0.457
Platelet count (10^3^/cm^3^)	Spearman’s rho *r*	−0.330*	−0.527**	0.608**
	*P*	0.027	< 0.0001	< 0.0001
Degree of thrombocytopenia	Point-biserial *r*	0.351*	0.250	0.418**
	*P*	0.018	0.097	0.004
Bleeding scale	Point-biserial *r*	0.360*	0.413**	0.453**
	*P*	0.015	0.005	0.002

*r* correlation coefficient; *MLKL* mixed-lineage kinase domain-like; *RIPK3* receptor-interacting protein kinase 3

**Correlation is significant at the 0.01 level (2-tailed)

*Correlation is significant at the 0.05 level (2-tailed)

Autophagy-related gene Beclin-1 mRNA expression levels negatively correlated with the age of the ITP patients (*r* = −0.406; *P* = 0.006) (Fig. 1f). Such correlation was not reported between the patient’s age and the mRNA levels of MLKL and RIPK3 (*r* = −0.12 and −0.199, respectively).

### Impact of Beclin-1, RIPK3 and MLKL mRNA expression levels on response to steroid therapy in ITP patients

Patients were grouped based on their response to first-line steroid therapy as responders, who achieved complete response criteria (*n* = 18, 40%) and non-responders, who did not achieve the complete response criteria (*n* = 27, 60%). The results demonstrated that the responders had significantly lower MLKL, RIPK3 and Beclin-1 mRNA expression levels (1.6 ± 0.28, 1.47 ± 0.13 and 1.92 ± 0.30, respectively) than their levels in the non-responders (4.44 ± 0.71, 3.8 ± 0.50 and 5.29 ± 0.59, respectively) at *P* < 0.01 as depicted in Fig. 2c.

**Fig. 3:**
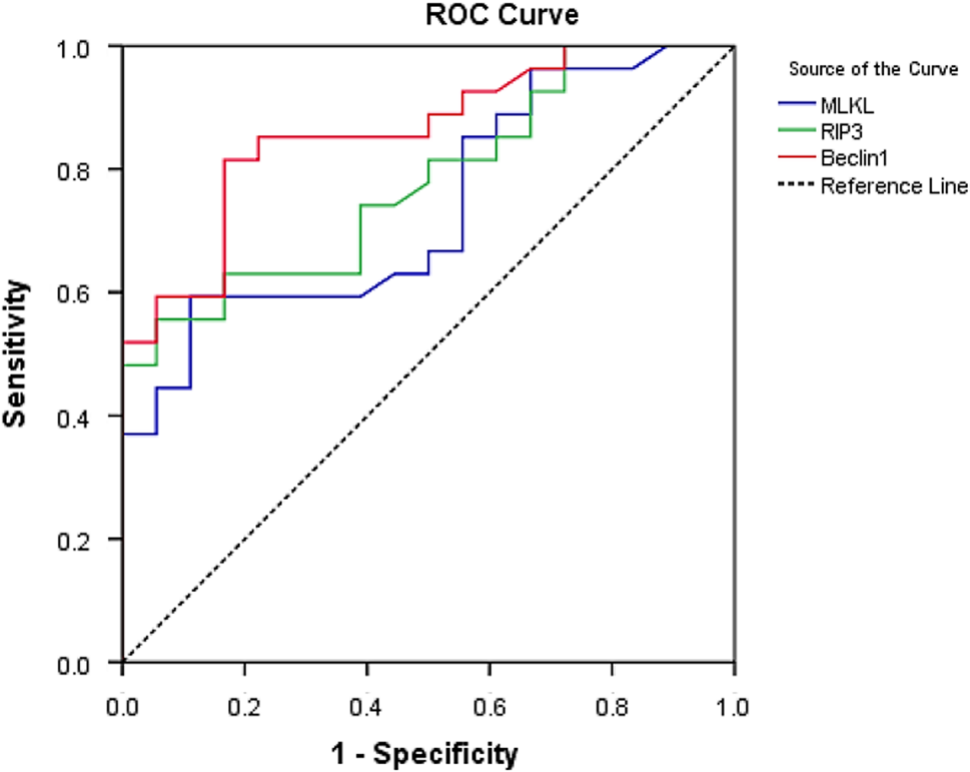
Receiver-operating characteristic (ROC) curve analysis for discrimination between steroid responders and non-responders in ITP patients using gene expression levels. MLKL, RIPK3, Beclin-1 mRNA expression levels at a cut-off value of > 1.973, 1.892, 1.912-fold, respectively, are good predictors for poor response to steroid (AUC: 0.739, 0.779, 0.861 sensitivity 59.3%, 62.9%, 85.2% and specificity 88.9%,83.3%,77.8%, respectively). MLKL, mixed-lineage kinase domain-like; RIPK3, receptor-interacting protein kinase 3

ROC curve threshold analysis was used to assess the sensitivity and specificity of the expression levels of both necroptosis-related genes (MLKL and RIPK3) and autophagy-related gene (Beclin-1) to discriminate between steroid responders and non-responder in ITP patients. Likely, MLKL, RIPK3, Beclin-1 mRNA expression levels at a cut-off value of > 1.973, 1.892, 1.912-fold, respectively, are good predictors for poor response to steroid (AUC: 0.739, 0.779, 0.861, sensitivity: 59.3%, 62.9%, 85.2% and specificity: 88.9%,83.3%,77.8%, respectively) (Fig. 3).

## Discussion

Despite recent breakthroughs in our understanding of ITP, the disease's pathophysiology is still not well understood [19, 20]. More research is needed to gain a better knowledge of ITP etiology that ultimately could lead to better diagnostic tools and novel predictors for bleeding severity, disease relapse, and treatment response as well as potential evidence-based new therapeutic modalities.

Dysregulated cell death pathways are closely related to the pathogenesis of ITP. However, the exact role of necroptosis and their crosstalk with autophagy in ITP as well as their link to patient’s characteristics and treatment outcomes are poorly investigated. This work explored the expression pattern of necroptosis-related markers MLKL and RIPK3 mRNA alongside autophagy-related protein Beclin-1 mRNA in adult ITP patients and highlighted the potential interconnection between necroptosis and autophagy in ITP patients.

The current study demonstrated that necroptosis-related markers (MLKL and RIPK3) are significantly overexpressed in ITP patients than in control subjects. Despite the lack of previous research discussing necroptosis in adult ITP to mention, there are studies exploring the involvement of necroptosis in different autoimmune diseases. Zhang *et al*., [38] reported upregulated MLKL mRNA expression in systemic lupus patients compared to healthy control and Duan *et al*., [39] who demonstrated upregulation of necroptosis markers (MLKL, RIPK1 and RIPK3) in psoriatic lesions.

The proposed mechanism of the upregulated necroptosis markers in ITP patients is related mainly to the defective clearance of necroptotic cells and release of their contents as DAMPs that induce sterile inflammatory response and release of cytokines such as type I and type III IFNs [40] leading to cytokine imbalance which is a form of the immune cellular dysregulations participating in ITP pathogenesis [41]. High levels of TNF-α, the major trigger of necroptosis, were detected in ITP patients [42, 43]. This indicates that necroptosis may provoke ITP through the initiation of inflammatory response and cytokines imbalance nevertheless necroptosis pathway may be triggered by the high TNF-α in ITP patients, creating a feedback loop in the progression of ITP.

The present work also reported significantly higher expression levels of autophagy-related protein, Beclin-1 mRNA in ITP patients compared to control individuals. Consistently, Liu *et al*., [44] observed that patients with active ITP had considerably higher Beclin-1 gene expression than healthy controls, highlighting the important role of autophagy in ITP. These findings were attributed to the fact that abnormally increased Beclin-1 expression will enhance autophagy of bone marrow megakaryocytes and inhibit their apoptosis with impaired platelet production leading to thrombocytopenia in ITP patients. On the other side, Shan *et al*., [45] explained that enhanced autophagy generally induces autoimmunity through enhanced survival and attenuated apoptosis of lymphocytes. They also suggested that platelet auto-antigens generated by protein degradation secondary to autophagy contribute to the ITP initiation.

To date, the interlink between autophagy and necroptosis is not fully clarified. Several studies have investigated such interconnection; however, the results were unfortunately controversial and conflicting [31, 32]. In this regard, the present study demonstrated a coincidental increase in necroptosis markers (MLKL and RIPK3) and autophagy-related protein Beclin-1 and reported statistically significant positive associations between Beclin-1 mRNA levels, and both MLKL and RIPK3 mRNA expression levels, supporting the suggested crosstalk between autophagy and necroptosis.

Some studies suggested that autophagy promotes necroptosis [46, 47] as a result of induced reactive oxygen species produced by autophagy and is considered one of the initiators of necroptosis [48]. On the contrary, Degterev *et al*., [49] reported that necroptosis stimulates autophagy as they demonstrated that necroptosis inhibitors can suppress the overexpression of Beclin-1, suggesting that autophagy is induced by necroptosis. On the other hand, other research demonstrated inhibition of necroptosis by autophagy [31] that is enhanced by relief of the inhibitory effect of mTOR signaling regulated by cellular metabolic and energetic status, emphasizing the autophagy's protective pro-survival effect against necroptosis [50].

Regarding clinicopathological characteristics of ITP patients, our results showed an inverse association between both necroptosis markers and Beclin-1 from one side and platelet count from the other side in ITP patients. Furthermore, the degree of thrombocytopenia was positively correlated with both MLKL and Beclin-1 expression levels. Patients with severe thrombocytopenia displayed significantly higher expression levels of MLKL and Beclin-1 than those who had moderate thrombocytopenia, indicating a causal role of both necroptosis and autophagy markers in the development of thrombocytopenia in ITP patients and confirming that ITP severity is driven by several mechanisms.

Severe bleeding is the most serious complication of ITP and a real challenge for disease management, concerning this and according to IBLS, our work revealed that ITP patients with severe bleeding had significantly increased levels of both necroptosis and autophagy markers than patients with mild bleeding in addition to positive correlations between these markers and severity of bleeding. This suggested that necroptosis markers (MLKL and RIPK3) and Beclin-1 may be novel biomarkers for the prediction of bleeding propensity and severity in ITP patients.

A remarkable finding of the present study is the significant low expression levels of both necroptosis markers (MLKL and RIPK3) and autophagy-related protein (Beclin-1) in ITP patients who respond to corticosteroid therapy than that in non-responder ITP patients. Moreover, we reported that MLKL, RIPK3 and Beclin-1 cut-off expression levels higher than 1.973, 1.892- and 1.912-fold expression, respectively, in ITP patients could detect the non-responders, this highlighting that MLKL, RIPK3 and Beclin-1 might be useful in predicting ITP patients that will benefit from steroid therapy.

## Conclusions

In conclusion, our work demonstrated the upregulation of necroptosis markers (MLKL and RIPK3) in ITP patients as well as their significant correlations with platelet count, bleeding severity and cortisol treatment response in those patients, implying that necroptosis may play a pivotal role in ITP pathogenesis. Our data also support the previously assumed role of autophagy in ITP pathogenesis and we disclose the crosstalk between necroptosis markers and autophagy-related protein Beclin-1 in ITP patients. Nevertheless, the interplay between the two processes is complex and not fully elucidated, necessitating further research.

## Limitations

The present study had few limitations. First, MLKL, RIPK3 and Beclin-1 expression were not detected in the bone marrow tissue. Second, differential expression patterns of target genes in megakaryocytes, platelets and other blood cell subsets have not been investigated. Third, the number of patients included may be relatively small.

## Recommendations

Based on our data, necroptosis markers may be promising therapeutic targets for ITP. A well-designed clinical trial to assess the efficacy of necroptosis inhibitors in the management of ITP patients is highly recommended, as are additional studies on a larger cohort of ITP patients to validate our suggestions for the use of MLKL, RIPK3 and Beclin-1 as promising markers for prediction of bleeding severity and treatment response in ITP patients. In addition, investigation of other cell death pathways such as pyroptosis may pave the way for a better understanding of ITP pathophysiology. Using high-throughput-based technology such as next-generation RNA-seq [51, 52] to gather more information about the megakaryocyte and platelet transcriptomes with regard to cell death regulating genes.

## Abbreviations

ITP: Immune thrombocytopenia
IBSL: ITP bleeding scale
MLKL: Mixed lineage kinase-like domain
RIPK: Receptor-interacting protein kinase
TNF: Tumor necrosis factor
